# Recommendations for the Application of Sex and Gender Medicine in Preclinical, Epidemiological and Clinical Research

**DOI:** 10.3390/jpm14090908

**Published:** 2024-08-27

**Authors:** Annamaria Cattaneo, Maria Bellenghi, Eliana Ferroni, Cristina Mangia, Matteo Marconi, Paola Rizza, Alice Borghini, Lorena Martini, Maria Novella Luciani, Elena Ortona, Alessandra Carè, Marialuisa Appetecchia, Ministry of Health-Gender Medicine Team

**Affiliations:** 1 Biological Psychiatry Unit, IRCCS Istituto Centro San Giovanni di Dio Fatebenefratelli, Via Pilastroni, 4, 25125 Brescia, Italy; 2 Department of Pharmacological and Biomolecular Sciences, University of Milan, 20133 Milan, Italy; 3 Center of Gender Specific Medicine, Istituto Superiore di Sanità, Viale Regina Elena 299, 00161 Rome, Italyalessandra.care@iss.it (A.C.); 4 Epidemiological System of the Veneto Region, Regional Center for Epidemiology, Veneto Region, 35100 Padova, Italy; 5 Istituto di Scienze dell’Atmosfera e del Clima, Consiglio Nazionale delle Ricerche, 73100 Lecce, Italy; c.mangia@isac.cnr.it; 6 Agenzia Nazionale per i Servizi Sanitari Regionali, 00187 Rome, Italy; 7 Ministry of Health, 00144 Rome, Italy; 8 Oncological Endocrinology Unit, IRCCS Regina Elena National Cancer Institute, Via Elio Chianesi, 00144 Rome, Italy

**Keywords:** sex, gender, preclinical, clinical, epidemiology

## Abstract

Gender medicine studies how health status and diseases differ between men and women in terms of prevention, therapeutic approach, prognosis, and psychological and social impact. Sex and gender analyses have been demonstrated to improve science, contributing to achieving real appropriateness and equity in the cure for each person. Therefore, it is fundamental to consider, both in preclinical and clinical research, the different clinical and biological features associated with sex and/or gender, where sex differences are mainly influenced by biological determinants and gender ones by socio-cultural and economic matters. This article was developed to provide knowledge and methodological tools for the development of studies/research protocols in which sex and gender should be taken into account.

## 1. Introduction

Sex and gender medicine represent a new dimension of medicine, looking at physiological and pathological differences between males and females; sex refers to biological and physiological characteristics and gender to the social constructions. This review aims to be a useful and concrete manual for those who want to include sex and gender determinants in their research protocol. Here, we first describe the main factors underlying sex and gender differences, and then we provide practical recommendations to consider in preclinical, clinical and epidemiological studies.

The need to include sex and gender assessment and consideration in medicine, healthcare and research is increasingly recognized [1,2,3]. Some aspects common to all research projects that want to consider sex and gender differences are listed below:

1. Formulate hypotheses on the effects that sex/gender may have on a given phenomenon included in the study;
2. Identify whether sex/gender differences have already been described in the literature;
3. Assess whether sex/gender should be considered as an independent variable, a modifier or a confounding factor and what the reason is;
4. Evaluate/calculate the statistical power of the sample size to be used in the analyses;
5. Collect and analyze sex/gender-disaggregated data;
6. Consider whether it is necessary to validate data in other studies using independent cohorts stratified for sex and gender;
7. Ensure that in the final scientific paper, the differences between sex/gender are reported in Tables and Figures and are used to obtain conclusions.

## 2. Factors Underlying Sex and Gender Differences

In the following paragraphs, we will discuss factors that can lead to sex and gender differences, including not only biological related factors (genetics, epigenetics, hormones, gut microbiome and immune/inflammation) but also social, environmental, cultural, ethnic factors, behavioral and psychological factors.

### 2.1. Genetic and Epigenetic Factors

Genetic mechanisms play a fundamental role in the differences between males and females. Genetic and epigenetic sex differences are correlated with the presence of two X chromosomes in females and a single copy of the X and Y chromosomes in males [3]. The Y chromosome hosts genes involved in male sex determination, cell cycle regulation, signal transduction, protein stability and the regulation of gene expression [4]. In “female” cells, one of the two X chromosomes is inactivated, although this inactivation may not be complete, as areas that escape inactivation exist; therefore, genes located in these areas may generate proteins expressed more in females than in males. Furthermore, many autosomal genes are differently expressed between males and females, further contributing to sex differences [5]. Also, epigenetic mechanisms can control gene expression through DNA methylation, histone modification, and also by noncoding RNAs or microRNAs. For example, more than 1000 CpG sites with different levels of methylation have been identified in the DNA of peripheral blood lymphocytes of men and women that can potentially contribute to sex differences in immune response via a methylation-mediated effect on mRNA levels of immune-related genes [6]. Moreover, the X chromosome contains a large number of microRNAs, about 120, which can thus be over-expressed in females if located in areas that escape inactivation, while only four miRNAs are found in the Y chromosome [4].

### 2.2. Hormonal Factors

Gonadal sex hormones. Gonadal sex hormones, such as estrogen, progesterone and testosterone, whose levels undergo fluctuations in different periods of life, induce important physiological changes in men and women [7]. Female sex hormones significantly change over time, with the modifications particularly relevant during puberty, pregnancy and menopause [8], differently influencing during the lifetime several physiological aspects, including those linked to cognitive and socio-emotional features and those associated with lipid and glucose metabolism, skeletal muscle, cardiovascular, hepatic, pulmonary function among others [9,10,11]. The immune response is also strongly influenced by sex hormones with estrogens that act as immunomodulators [12]: at high levels, such as those observed during the follicular phase of the menstrual cycle or in pregnancy, they have an anti-inflammatory effect, while at lower levels, such as those found in other phases of the menstrual cycle, have a pro-inflammatory effect [13]. It is also necessary to consider the role of some environmental or food endocrine disruptors and of hormonal, contraceptive or replacement therapies, which can have a sex-specific pathophysiological effect [14,15,16]. Likewise, aromatase inhibitors, blockers of estrogens, and inhibitors of the enzyme 5-alpha-reductase types I and II can cause important sex-specific side effects.

Stress hormones. Cortisol, the stress hormone, if produced for a long period as a consequence of exposure to stressful events, can have negative effects on health by increasing the risk for mental disorders, such as depression and anxiety, and for metabolic and cardiovascular disorders. Literature data report that women are at higher risk of these disorders than men [17]. In general, our body is capable of reacting to stress, but in the long term, chronic stress can trigger serious pathologies. Indeed, it is well known that males and females respond in different ways to the same stressful stimuli. Chronic stressors in women are more frequently associated with an enhanced risk of depressive symptoms, while men show an increased risk for addiction or cardiovascular-related disorders. In a large-scale prospective analysis conducted in an adult population, it has been observed an association between stress and an increased risk of developing psychiatric diseases, such as anxiety and depression, but also metabolic and cardiovascular diseases [18,19,20]. It has been stated that depression is twice as frequent in women as in men, and it occurs frequently in comorbidity with cardiovascular diseases [21,22]. It is known, in fact, the positive correlation between depressive disorder and acute myocardial infarction, heart failure or stroke. In addition, anxiety, another cardiovascular risk factor, occurs more frequently in women later in life, with a ratio of men to women of 1:1.7, and correlates with acute coronary syndromes in patients [23] with coronary artery disease [24,25]. Takotsubo cardiomyopathy (TS), characterized by transient contractile dysfunction of the left ventricle, is the result of intense psychological stress that causes the release of circulating catecholamines and altered coronary vasodilation. Reduced estrogen rates that occur during the postmenopausal period increase the susceptibility to develop Takotsubo syndrome, and it has been shown that women over the age of 55 are five times more likely to develop TS than younger women [26]. Mental stress can induce more negative emotions in women than men and trigger greater platelet aggregation and more cases of myocardial ischemia [27]. In addition, stress response can also influence responses to vaccines and modify the pharmacokinetics and pharmacodynamics of different drugs that consequently can be different between men and women [28].

Another difference between men and women in relation to stress-related hormones concerns the production of adrenaline, a hormone that triggers typical fight-or-flight reactions. Its levels increase physiologically in both sexes, but in males, a positive correlation between motivation for success and a negative one with anxiety has been observed, while in females, they are associated with low self-esteem and a lack of satisfaction with social expectations [29]. Together with adrenaline, also the level of cortisol increases: in males, this hormone reaches levels that are almost double those observed in females. Moreover, males show an increase in anticipation of stressful events, which does not occur in females [30]. The hypothalamic–pituitary–adrenal (HPA) axis, the main component of the neuroendocrine system, works together with the Hypothalamo–Pituitary–Gonadal (HPG) axis to finely regulate environmental, psychological, reproductive, and genetic factors that respond to internal and external stressors. Proper functioning of both HPG and HPA axes is fundamental for maintaining physical and mental health since their alterations have been associated with several mental and physical disorders [31]. Recent literature reviews [31,32] support the involvement of sex-specific differences in the regulation of the HPG and HPA axes in response to stress, and it has been suggested that these differences may partly explain the prevalence of the female sex in stress-related mental disorders. Preclinical models have clearly demonstrated that the HPA axis in females is more quickly activated, resulting in greater production of cortisol as compared to males. Indeed, the fluctuating levels of steroid hormones in females during the menstrual cycle are known to contribute to sex differences in the HPA axis activity therefore influencing also the response to stressful stimuli. However, several clinical studies have produced conflicting results, probably due to different types of stressful events, the age of the participants, the use of contraceptives and the different phases of the menstrual cycle. Further studies are needed to delve deeper and clarify the mechanisms underlying the different responses to stress in males as compared to females [32], and in this context, it will be important to consider the contribution of several other factors, such as luteinizing hormone (LH), follicle-stimulating hormone (FSH) and aldosterone [31].

Taking all this into account, studies that evaluate the impact of stress should consider the role of endogenous and exogenous hormones on the different parameters, also collecting data on the menstrual cycle or the fertility age and performing replication studies to validate the results obtained. It should be finally underlined that not all sex differences in stress response and associated vulnerability are linked to hormones, as some differences are manifested already during the early stages of prenatal life, before the appearance of sex hormones [33].

### 2.3. Microbiota Composition and Metabolites

The term human microbiota refers to the combination of microorganisms (bacteria, archaea, protozoa, fungi and algae), mainly present in the intestine, which, in physiological and sometimes pathological conditions, live in symbiosis with the organism [34]. The microbiota is a constantly changing system, and it is influenced by several factors such as age, lifestyle, environment, diet, body mass index (BMI), drug therapies, including hormones, ethnicity and even sex and gender [35]. Although differences in the gut microbiota composition between men and women are poorly understood [36], it appears that these differences occur during puberty and are associated with the changes in sexual hormone levels that characterize this period [37]. However, some authors have demonstrated that sex differences can already be observed in the microbiota of premature babies [38] and are mainly found in women during particular periods of life, such as pregnancy [39]. Importantly, the literature also reports that the microbiota is closely connected with the immune system and that differences between men and women in its composition can shape, again in a sex-dependent manner, immune responses [40]. Recent scientific evidence has also demonstrated that the intestinal microbiota is interconnected with the functioning of the central nervous system, shaping cognitive development and some emotional-behavioral features. In this context, sex could play a fundamental role in the development of several psychiatric or neurodegenerative diseases [41]. Considering all this evidence, it is essential to understand how sex can influence the composition of the intestinal microbiota, which, in turn, affects the immune response and the functioning of the central nervous system. However, to date, studies investigating the impact of the gut microbiome composition on physical and mental health, which dissect the data by sex, are still limited. Therefore, future preclinical projects should study the composition of the microbiota in animal models that include both sexes in the same numerical ratio. Similarly, studies planning patient-to-animal fecal transplantation should be performed by having the patient and the transplanted animal of the same sex. Finally, clinical studies, including trials, should not only show data divided by sex but also consider the different influences of gender-specific behavioral factors, lifestyle and dietary habits on the composition of the microbiota in males and females.

### 2.4. Immunity and Inflammation

Many studies suggest that specific factors associated with sex are essential in regulating immune responses and inflammatory processes. Indeed, the immune system works in different ways in men and women. While men are generally more susceptible to pathogen infections, women are more frequently affected by autoimmune diseases and have a higher immune response to vaccines [42,43]. These differences in inflammatory/immune response can depend on different factors, including lifestyle habits, environment, hormonal factors, and genetic and epigenetic factors. As anticipated, estrogens may have anti-inflammatory or pro-inflammatory activities depending on their levels [44], in turn influencing the immune responses. For example, pregnancy is well known to represent a critical period for the onset and course of autoimmune diseases and changes in hormone levels that characterize this period have an influence on disease symptoms [44,45]. Considering this, it becomes extremely important to consider variables, such as the phase of the reproductive cycle as well as fertility (e.g., pregnancy, menopause) or even the cycle phase in preclinical studies, in all the different phases of research (project planning, data analysis, data collection, results description and discussion of results) both in preclinical and clinical studies.

### 2.5. Environmental, Socio-Cultural, and Ethnic Factors

Some of the differences between men and women are social and cultural in nature and contribute to shaping the concept of gender. While with “sex” we refer to features that are biologically determined, gender roles are acquired after learning and adopting different behaviors, which are influenced by the social environment. Gender role is indeed acquired during the socialization process, and it comes from the interrelationship between parents’ attitudes, the education received and the sociocultural environment [46]. In addition, gender behavior is not a dichotomous variable, but it is rather defined by behavioral, psychological and cultural factors that are expressed on a continuum. Therefore, gender appears to be complex and multidimensional, not easily associated with variables and features that can be analyzed in the scientific field without the collaboration of experts in social sciences.

Importantly, gender attitudes and behaviors promote different patterns of healthy or unhealthy lifestyles among women and men [47]. Considering this, it is quite easy to understand the reason why different and inequitable exposure to health risks that can be gender-related can have an important impact on health outcomes. For example, it has been demonstrated that low socioeconomic status has a stronger effect on women than on men [48]. Among women, several chronic diseases, such as cardiovascular diseases, type 2 diabetes mellitus, cancers, and chronic respiratory diseases, are the most significant cause of death in low- and medium-income countries, especially during childbearing.

It becomes essential when drafting a research project or a clinical study to consider all the factors that can influence gender differences at various levels. Future studies, in addition to recruiting men and women as balanced as possible, will have to take into account the opportunity to include representatives of the different ethnic groups present in the national territory.

### 2.6. Behavioral and Psychological Factors

The gender dimension is closely connected to models and attitudes linked to expectations and social relationships [49], influencing the quality of access to healthcare and the right to live a healthy life, such as relationships with doctors and healthcare facilities or different ways of internalizing a diagnosis [50]. However, in everyday medical practice and in the subjective experience of health and illness, it is not easy to separate the influence of sex from that of gender. Sex-related factors can influence health by affecting behavior: testosterone, for example, can cause aggressive reactions associated with exposure to risk and failure to comply with medical recommendations. Furthermore, gender behaviors can modify biological factors and, therefore, health status: exposure to stress, toxins, nutrition or lifestyle choices can induce genomic and long-lasting modifications due to changes in epigenetics in adults and children [51]. The most interesting peculiarity of an epigenetic modification is that it can take place in response to external environmental stimuli, which concern not only the environment but also gender-determined behaviors. To note, epigenetic marks are reversible, but can also undergo inheritance [52].

Literature data reported statistically significant psychological differences between men and women. In relation to cognitive aspects, women seem to be more competent in linguistic-verbal skills, non-verbal communication and the expression and recognition of emotions, whereas men would be more skilled in visuospatial tasks, such as orientation and mental rotation of objects [53,54]. Taking behavioral aspects into consideration, women express their emotions more openly, have strong relational temperaments, show greater empathic capacity and are inclined to ask for help more easily if experiencing physical and mental health problems [55,56]. In relation to mental health, some disorders, such as depression and anxiety, are more represented in females; moreover, depressive and anxiety symptoms show a symptomatic picture and a different course in the two sexes [57]. It is, therefore, essential when planning research projects to have clear knowledge of these aspects to propose adequate psychological evaluation tools, with the final aim to consider sex and gender differences during clinical evaluations, removing any type of stereotype that sometimes considers women more vulnerable and fragile than men [58]. From a gender medicine perspective, it is important to engage researchers, clinicians and psychologists to create a multidisciplinary team dedicated to the development of protocols for sex and gender-specific diagnosis, evaluation and rehabilitation.

## 3. Preclinical Studies

Preclinical studies represent a fundamental tool to identify pathogenic mechanisms, but also diagnostic, prognostic and predictive biomarkers and/or therapeutic targets. In addition, they are useful in the assessment of new pharmacological compounds or medical devices before they are translated into humans. Preclinical studies are based on different models: (i) in vitro, e.g., cells isolated and grown in the laboratory; (ii) ex vivo, e.g., freshly isolated material/tissue or cells/blood from human volunteers; and (iii) in vivo, e.g., laboratory animals [59,60]. More recently, in silico models have also been developed, where computational simulations are used to mimic and reproduce complex pharmacologic or physiologic processes [61]. Moreover, during the last years, to overcome the requirements associated with in vivo preclinical models, alternative approaches, such as tissue-specific bioreactors, three-dimensional (3D) organoid cultures, and organ chips, have also been developed [62]. Nonetheless, traditional in vivo animal models and in vitro studies are still considered necessary for the advance of innovative medical approaches. Furthermore, as the interest in sex and/or gender differences has grown significantly, female and male disparities should be considered in all the preclinical studies. However, in most cases, published data do not state the sex of the organisms from which the stabilized cell lines or freshly isolated cells have originated [63,64]. Regarding animal models, most of the published works are focused on male animals only, although recently, data comparisons between males and females have been requested [65,66].

### 3.1. In Vitro and Ex Vivo Studies: Cells Have a Sex

In vitro studies mostly utilize cell lines kept in culture for decades and are no longer representative of their original sex, considering that experimentally cultured cells remember their original sex only for a short time. It is well known how passages and time in culture can lead to accumulating changes, including karyotypic alterations and loss or gain of chromosomes [67]. As a result, several studies have shown that the same cell line can be different and produce different data from one laboratory to another. The American Type Culture Collection (ATCC) has reported that, in their collection, about 100 cell lines, originally derived from men or male mice, have “lost” their Y chromosome, a phenomenon that is particularly common in cancer cell lines. Chromosome instability is particularly common in immortalized cell lines: cells transformed by viral oncogenes frequently accumulate major changes, including loss of both autosomal and sex chromosomes. Importantly, these changes occur randomly, and cultures of transformed cells tend to have a mosaic of chromosome patterns. Therefore, as an overall consequence, transformed cell lines do not allow the evaluation of sex as a biological variable.

When working with isolated cells, it is important to consider the sex of the donor since it is well known that different types of male and female cells (e.g., endothelial cells, smooth muscle cells, etc.) respond differently to chemical stimuli and alterations of the microenvironment due to genetic and phenotypic intrinsic differences [63,68]. Among the differences underlying the onset and progression of many diseases, we can mention the mechanisms associated with the different responses of XX and XY cells. Indeed, when exposed to stress stimuli, female cells are able to implement protective mechanisms, such as autophagy and antioxidative processes, while male cells, under the same stressors, more easily die undergoing apoptosis [69,70]. This diversity in activating different biological mechanisms in response to similar stressors might also depend on the different efficiency of the detoxification system, as some male cellular types have lower antioxidant properties, for example, lower levels of glutathione. Interestingly, the sex-specific behavior is lost after 7–10 passages, and cells become all [69]. Since the use of cell lines represents an indispensable tool for all preclinical laboratory studies, from the “screening” of new drugs to toxicity evaluations, it is essential to use cells rigorously controlled after isolation and kept in culture for a short time. To conclude, to unveil any possible relevant sex difference, it is needed to compare male and female cell lines and, when possible, also to prepare a database with the features of each donor in terms of gender, sex and age [71].

### 3.2. Cell Cultures and Media

“Cell culture medium” is a general term that includes any liquid created to support cellular growth in an artificial setting. A typical culture medium contains a balance of amino acids, vitamins, inorganic salts, glucose, and serum as a source of growth factors and hormones. In addition to nutrients, the medium also contains substances that help to maintain pH and osmolality. Different cell lines have different nutritional requirements, thus requiring media optimization methods. A comparison between the most used media, such as Dulbecco’s Modified Eagle Medium (DMEM) and Roswell Park Memorial Institute 1640 (RPMI1640), and normal human or murine plasma revealed significant differences [72]. Furthermore, many culture media contain estrogen-like factors able to bind estrogen receptors (ERs), mimicking the hormone actions in influencing proliferation, differentiation and cell metabolism [73]. It is then important to consider the possible confounding factors, such as sex hormones and growth factors, which, present in the culture media, can modify the results.

Here are discussed three main components of the media.

Animal serum. Commercial cell culture media to increase cell vitality often require the addition of animal sera, which contain hormones, including sex steroids, proteins, lipids, carbohydrates, vitamins and growth factors. Fetal bovine serum and fetal calf serum are the more commonly used [74]. Actually, adding serum to the media might pose some problems: (i) serum composition is not ever exactly the same, depending on the mother’s age, the composition of the feed, the slaughter season and the geographical location of the slaughterhouse; (ii) FBS batches are often prepared by combining sera from several fetuses with the idea that using these pools would minimize variability. As timing and costs often do not allow considering differences in serum batches, researchers also try to reduce concentrations of sex hormones by using charcoal-filtered sera. As differences have been observed between batches of Charcoal Stripped Fetal Bovine Serum, it is important to indicate brand and lot number, possibly using the same one for performing experiments included in the same study [75,76].

Phenol red. Phenol red, a common pH indicator found in most commercially available media, appears bright red in cell cultures at pH 7.4 and becomes yellow during acidification of the media associated with cell growth. Commercial media contain different concentrations of phenol red, going from 40 µM in DMEM to 22 µM and 13 µM in DMEM/F12 e RPMI 1640, respectively. Phenol red is similar in structure to some nonsteroidal estrogens, and it is able to bind and activate ERs [77]. It is then important to consider that phenol red might have per se some effect on cell lines, including tumor cells, and that serum can bind phenol red in turn reducing its activity. In this regard, it has been shown by using the MCF7 ER+ breast cancer cell line that the presence of higher proliferation is associated with high levels of phenol red [76,78]. In addition, primary cultures of normal cells (immature pituitary cells and rat uterine cells) show increased expression of biomarkers associated with proliferation and differentiation in the presence of phenol red. Like 17 β-estradiol (E2), phenol red inhibits neuron depolarization [78]. Once again, researchers should take in mind the confounding effects possibly associated with phenol red, indicating the brand and batch number of the cell cultures. Commercially available media without phenol red are useful for testing its possible influence on the parameters to be analyzed [79].

Plastic ware. The plastic used for most cell cultures contains polystyrene, which releases into the medium phenolic compounds, acting as weak estrogens competing with exogenous estrogens. Thus, even the plastic ware should be considered as a possible estrogenic confounder [80] and researchers are encouraged to look at possible plastic-associated differences in estrogen-dependent cells.

### 3.3. In Vivo Studies on Experimental Animals

In the United States, the Food and Drug Administration and the National Institutes of Health approximately ten years ago provided suggestions for a proper design for preclinical models, which still represent an essential tool for studying the efficacy of different interventions [81]. However, we are far from having a balanced number of male and female animals included in preclinical studies as, in most cases, just one sex, usually the male one, is evaluated, and only a few experiments and papers, approximately 10%, enroll both males and females [82]. Studying animals with the same genetic background but reared in different environments can introduce confounding non-sex-related differences in offspring; therefore, when comparing males and females, it should be better to have them as littermates [81,82]. Further attention should be paid to possible exogenous sources of estrogens, such as rodent food (soy phytoestrogens), bedding (cob), cages and water bottles (plastic bisphenol-A) [80,83]. Other aspects to consider are represented by the season and the time of the experiment, which could lead to possible hormonal fluctuations. Testosterone, for example, fluctuates seasonally, with circadian rhythms, and the concentrations of female hormones can fluctuate even over the course of a single day due to the estrous cycle. Therefore, it should be important to record the photoperiod in the colony, and the time during the day the measurements are taken. Finally, also the researchers’ sex, could possibly impact the rodents’ responses to pain. For example, the presence of a male researcher has been associated with a reduced pain response as compared to a female researcher, both for male and female rodents [84].

### 3.4. The Role of Gonadal Hormones after Puberty in Animal Models-Based Studies

Looking at sex-differences in rodents, the roles of gonadal hormones such as E2 and progesterone in females and testosterone in males should be mentioned [85]. To answer these questions, oophorectomy (OVX) experiments in females to suppress ovarian secretions, including E2 and progesterone (P4), and orchiectomy experiments in males to determine the impact of testosterone have been performed. Since surgery causes long-lasting changes in the hypothalamic–pituitary–adrenal axis and affects stress hormones, it is essential to expose the control group to sham surgery [86]. An example of a proper experimental design looking at E2 function usually includes three groups: (i) control animals with intact gonads, (ii) OVX with medium plus E2 administration and (iii) OVX with medium administration without the addition of E2. If E2 can restore the phenotype to the level of controls, the conclusion can be that E2 contributes to the observed sex difference. Several methods of E2 replacement therapy can be used, including daily subcutaneous (SC) injection of E2 in oil or SC implantation of commercially available E2-containing pellets. Once the role of E2 is confirmed, it may be important to determine which estrogen receptor (ER) mediates the effect. This can be evaluated by using commercially available agonists for ERα (1,3,5-tris (4-hydroxyphenyl)-4-propyl-1H-pyrazole- PPT) or for ERβ (2,3-bis (4-hydroxyphenyl) propionitrile-DPN), but a definitive confirmation can be obtained in female mice KO for these receptors.

The so-called “Four core genotypes” (FCG) murine model allows us to dissect the roles of sex chromosomes, gonadal hormones or the involvement of both groups of factors. The FCG model includes XX gonadal males or females, and XY gonadal males or females. This model consists of transferring the Sry gene from chromosome Y to an autosome to obtain XX and XY mice with either ovaries or testes. These mice, uncoupling the roles of sex chromosomes from those of sex hormones, allow us to identify the specific effects on cell and tissue phenotypes of chromosomes, gonads or their interactions [87]. In particular, this model allows us to evaluate the differences in phenotypes caused by (i) sex chromosomes (XX vs. XY), (ii) sex hormones (ovarian vs. testicular hormones), and (iii) the interactions of sex chromosomes and sex hormones. When a sex chromosome effect is found, it could be possible to evaluate the X or Y genes that could be responsible for this effect by comparing XO with XX and XY after oophorectomy or orchiectomy to eliminate sex hormone influences. When the effect in XO mice is comparable to that observed in XY and diverse to that of XX mice, we can conclude that the observed effect depends on the number of X chromosomes. On the other hand, if the effect in XO and XX mice are similar but different to that observed in XY it is possible to assume that Y-linked genes cause the sex chromosome effect.

For further details, see the document “European Commission, Directorate-General for Research and Innovation, Gendered innovations: how inclusive analysis contributes to research and innovation” [88].

## 4. Epidemiological Studies and Issues from a Sex and Gender Perspective

For a long time in medical research, it has been considered that male and female bodies differ only in size and reproductive physiology, effectively assuming a hypothetical male standard from which women escaped due to certain characteristics. Epidemiology has played a key role in bringing out differences between males and females in the development, symptomatology, and prognosis of diseases, shifting the paradigm of a male standard towards the idea of sex and gender-specific medicine. Indeed, males and females do not become sick in the same way: both incidence and prevalence of diseases are different in the two sexes and, consequently, their impact in terms of health care resource use is also different [89]. Despite these differences, the variable “sex” is not always considered appropriately in epidemiological studies; analyses are often presented reporting overall data, which includes both men and women, not giving any possible sex difference a chance to emerge [90]. Sex should be taken into consideration not only in the results but also in the study design [91]. This aspect is very important, especially in the field of pharmaco-epidemiology studies, as they evaluate the effectiveness and safety of specific pharmacological treatments. Women, however, are overall under-represented in randomized clinical trials: the percentage of women enrolled hardly exceeds 20% in phase 3 clinical trials, and, as a result, in some cases, drug approval is based on data collected mainly from the male population [92,93].

### Sex and Gender Dimensions in Epidemiological Studies

Stratification by sex is also important for a careful understanding of the population’s health status. While stratification by sex in epidemiology is an easy procedure to perform, considering the variable “gender” represents a more complex issue. In epidemiological studies, there is often confusion between the dimensions of sex and gender, and often the two terms are used interchangeably. By confusing gender categories with sex, researchers not only risk undermining the validity of gender analysis but also limit the understanding of how relevant the two types of factors can be, with implications for both treatment and prevention. Although including sex and gender in research designs became a requirement for many researchers and funding agencies [94], no standardized measurements of gender have been implemented so far, and many researchers are still struggling with how to operationalize the variable “gender” in their analyses. The Canadian Women’s Health Research Network has identified four domains that encompass gender: gender identity, gender roles, gender relations, and institutionalized gender. Among these gender dimensions, some gender-related variables known to have an impact on health were identified (i.e., occupation, marital status and wage gap). The selection of specific gender-related variables should be guided by the initial research question and should be based on their relevance to the study in order to proactively incorporate gender into the research [94,95].

The collection of data and information, the questionnaires underlying the surveys, the variables relevant to the study and the presentation of results require attention to sex and gender. In many studies, for example, results are stratified by classes more representative of working life rather than by physiological evolutions in the lives of men and women. Consider, for example, how the menopause-related age group is also neglected when analyzing cardiac outcomes despite the known protective effects on the cardiovascular system of female hormones [96,97]. However, an obvious gender bias also exists in the definition of working life itself, which considers only paid service and unpaid domestic work as such and does not recognize as occupational factors the risks to which women are exposed inside the home (indoor pollution, domestic accidents, etc.). In addition, the increased time spent in unpaid domestic work leads women in many areas to be more settled and likely to be more affected by any contamination present in their area of residence. These aspects, especially in environmental epidemiology studies, may lead to underestimation of environment-related health problems in one or both genders, with the consequence that those problems may be attributed to other causes and not addressed in public health policies. Therefore, in these areas, the study design needs to ask whether (i) the initial research questions adequately capture the exposure of men and women, (ii) physiological differences have been taken into account in the choice of biomarkers, (iii) the choice of health outcomes is appropriate for both genders; (iv) the public health claims affect both genders similarly [98,99,100]. In conclusion, epidemiological research from a sex and gender perspective requires an awareness of how men and women, in addition to being different physiologically, have different social and cultural roles. These are intertwined with the biological datum in varying ways throughout life and are subject to conditioning and prejudices, which have an impact on the health status of the two sexes, consciously.

## 5. Sex and Gender Pitfalls in Clinical Studies and Trials

Over the centuries, women have been excluded or under-represented in clinical trials, as they have been considered inappropriate due to some variables, including those related to the hormonal cycle [93]. As a result, women could receive misdiagnoses, miss treatment opportunities, receive inappropriate doses of medication, or even receive “wrong” drug prescriptions [101]. This has reduced the possibility of generalizing the results from clinical trials to the entire population. Over the years, there has been a strong correlation between sex and incidence, prevalence, symptoms, age of onset and severity of some pathologies [102,103], as well as a different response to drugs [104]. Preclinical and clinical studies have shown that there are differences based on sex at the genetic, cellular, biochemical and physiological levels [105]. Therefore, it is now reasonably clear that the results from clinical trials on drug safety and drug efficacy would be more scientifically truthful if they included women and men equally so as to exploit the knowledge to improve diagnosis and pharmacological treatments for their respective specific target of patients [105]. Over the past 30 years, efforts have been made to increase the presence of women in clinical trials. Nevertheless, the results of the study by Steinberg and collaborators suggest that this sex-related bias still exists, at least in North American studies [106]. Indeed, analyzing about 20,000 clinical trials published from 2000 to 2020 in oncology, neurology, immunology and nephrology, women were reported to be still underrepresented [106,107]; on the contrary, studies focused on preventive interventions showed a greater representativeness of women. An additional problem, still related to the low presence of women in clinical trials, is the low number of studies aimed at obtaining detailed results based on sex or gender-related features. The study by Sugimoto and collaborators [105], analyzing articles published between 1980 and 2016, found that the percentage of public healthcare studies containing explicit sex-disaggregated analyses grew from 36% to 69%. However, the increase was much lower in clinical trials (from 59% to 67%) and in laboratory-based research, where sex-disaggregated analyses are still underrepresented (31% in 2016). Due to the lack of adequate planning of clinical trials, providing a priori analyses that consider the differences resulting from biological attributes rather than from roles, identities and socially constructed behaviors of women and men, it is very difficult to extrapolate the results obtained from the analysis of aggregated data [108]. Regarding the general principles to be followed in the planning and processing phases of clinical trial data, we can rely on the work of Tannenbaum and collaborators [108], on the work of McGregor and collaborators [109] and on the SAGER guidelines [110].

### Sex-Specific Biomarkers

A biomarker is, by definition, a biological, genetic, or biochemical marker that can be measured in human tissues, cells, or fluids (such as serum, urine, and saliva) and that can be related to the onset or development of a disease, or to the efficacy or adverse events of pharmacological or non-pharmacological interventions [111]. Biomarkers can be used individually or in combination with others to improve their properties/roles (e.g., biomarkers as predictors of response or of the onset of side effects).

Practical examples come from genomics, where multi-genes, called genomic “signatures”, are often used to predict a specific clinical outcome [112]. Generally, one individual biomarker is not able to be diagnostic, prognostic, and predictive simultaneously, and biomarkers are, in general, pathology- and treatment-specific [111]. There are also exceptions in oncology; for example, Ki-67 has a prognostic but also a predictive role, and it is used together with clinical parameters, such as stage, grade of the tumor and endocrine status for the choice of systemic treatment of breast cancer. Moreover, there are genetic biomarkers associated with prognosis for different types of tumors [113,114].

The identification of biomarkers is a complex process, and the possible effects induced by different confounding variables, such as environmental factors, not often considered in experimental design or data analysis, should always be taken into consideration [115]. In addition, other variables should be considered, such as the time of collection of the biological sample, the time spent between collection and measurement, the time from food intake, the place of residence, age, smoking and alcohol habits, and many other factors [116]. It should also not be forgotten that some characteristics of biomarkers may be sex-specific, meaning that some markers may have a diagnostic, prognostic or predictive value in one sex but not in the other [117,118], and this can only be deduced by analyzing the data disaggregated by sex. Furthermore, it is essential to also consider the different periods of life, particularly for women, such as puberty, fertile period, perimenopause and menopause, which are characterized by important hormonal fluctuations that can influence the predictive value of a biomarker. Biomarkers should have optimal operational features and high predictive value in a differential way between women and men to detect pathologies early, recognize patients who will have a poor prognosis due to the disease, and allow the development of customized treatment plans [119,120,121,122]. In cardiovascular diseases, in terms of disease prediction, it has been observed that cholesterol has a greater predictive power in men than in women, while a combination of arterial hypertension and diabetes mellitus seems to be the main predicting factor in women [123]. Sex differences in the levels of certain markers in the blood have also been observed in neurodegenerative diseases. For example, the alpha-synuclein biomarker for Parkinson’s disease, in addition to being present in the CSF and whose levels are correlated with the severity of the disease, may also be measurable in the blood (plasma) where significantly different values have been reported between men and women, and which could be linked to the protective power of hormones in women [124]. Another example of sex-specific biomarkers, in this case of genetic nature, can be found in Amyotrophic lateral sclerosis (ALS), where, for example, some polymorphisms in the MTHFR gene seem to increase the risk of disease in women [123]. Another interesting example of sex-specific biomarkers is reported in a recent study by Pagano and collaborators [118]. In this study, male and female patients with COVID-19 at the time of hospitalization were assessed for measurements of several circulating markers (ferritin, D-Dimer, neutrophil and lymphocyte counts, testosterone, soluble ACE2 estradiol and angiotensin 1–7), in order to evaluate their predictive value of the onset of acute respiratory syndrome during hospitalization. All the markers were able to predict the onset of respiratory syndrome when analyzed together in a neutral manner, but when the data were disaggregated by sex, high neutrophil counts and low levels of angiotensin 1–7 were able to predict respiratory syndrome only in women and not in men. In contrast, low lymphocyte counts and low levels of circulating testosterone resulted as predictors of respiratory syndrome exclusively in men. The other markers maintained predictive value in both sexes. It is, therefore, evident the need to evaluate the sex-specificity of the markers used for clinical appropriateness and scientific rigor.

## 6. Discussion

Sex and gender equity in general health are well recognized to represent a global priority. To apply the sex and gender issues in preclinical, clinical research and therapeutical approaches, multidisciplinary methods that take into consideration all the biological, social and economic factors that can lead to health differences should be developed and then applied.

Indeed, the new dimension of sex and gender-specific medicine represents a strategic issue for public health. It is important to underlie that sex and/or gender differences should be included in all the studies, going from basic to translational research, covering preclinical models and clinical studies and trials. We know that differences between the sexes have been found in all major groups of pathologies potentially affecting both sexes and that they arise from a combination of environmental, genetic, and epigenetic factors, as well as differences in gene regulation and expression. Precision and tailored medicine, whose relevance is steadily growing, cannot fail to include sex and gender differences to effectively consider disparities and identify the best therapeutic action for each individual person. Nonetheless, we are far from balanced inclusions of female and male animals and women are still significantly underrepresented in clinical trials. An important advancement was made in 2020, when the European Commission requested in the Horizon Europe program the inclusion of gender analysis in the design of research, thus encouraging the application of gender medicine in research. Very important was also the inclusion in the instructions for the authors of many scientific journals, including those of the publishing group Nature and Lancet, the request to include sex and gender among the parameters under analysis in their study. In particular, it is requested to specify whether the results set out in the article apply uniformly to women and men or, conversely, gender differences have been highlighted. The rationale behind these requirements is based on the SAGER (Sex And Gender Equity in Research) guidelines of the Gender Policy Committee of the European Association of Science Editors (EASE). The guidelines are aimed at scientific journal editors and authors with the aim of including sex and gender determinants in their research so that data can be analyzed in both sexes and authors can present disaggregated data. Another fundamental step in Italy was represented in March 2023 by the inclusion of sex and gender differences in the methodological standards for the development and updating of guidelines in clinical practice, published on the website of the National Guidelines System.

## 7. Conclusions

Overall, it becomes essential when drafting a research project or a clinical study to consider all the factors that can influence sex and/or gender differences at various levels. Future studies, in addition to recruiting men and women as balanced as possible, will have to consider the opportunity to include representatives of different ethnic groups present in the national territory.

In conclusion, applying a gender approach in research and treatment pathways is important not only to improve the understanding of health and disease determinants in the broader sense but also represents a key link towards greater equity of access to care and medicine increasingly focused on the characteristics of the patient helping to strengthen the “centrality of the person”. Gender medicine should, therefore, be understood as a strategic objective for public health.

## Data Availability

Not applicable.
